# Lung Metastases From Papillary Thyroid Cancer With Persistently Negative Thyroglobulin and Elevated Thyroglobulin Antibody Levels During Radioactive Iodine Treatment and Follow-Up: Long-Term Outcomes and Prognostic Indicators

**DOI:** 10.3389/fendo.2019.00903

**Published:** 2020-01-10

**Authors:** Zhong-Ling Qiu, Chen-Tian Shen, Zhen-Kui Sun, Hong-Jun Song, Guo-Qiang Zhang, Quan-Yong Luo

**Affiliations:** Department of Nuclear Medicine, Shanghai Jiao Tong University Affiliated Sixth People's Hospital, Shanghai, China

**Keywords:** papillary thyroid cancer, lung metastases, elevated thyroglobulin antibody, radioiodine therapy, negative thyroglobulin

## Abstract

**Background:** The lung is the most frequent site of distant metastasis from differentiated thyroid cancer (DTC). However, lung metastasis from papillary thyroid cancer (PTC) with persistently negative thyroglobulin (Tg) and elevated Tg antibody (TgAb) levels is an extremely rare entity, and the prognosis is therefore elusive. We investigated the clinical characteristics, long-term outcomes, and prognostic factors of lung metastases in PTC patients with persistently negative thyroglobulin (Tg) and elevated Tg antibody (TgAb) levels during radioactive iodine (^131^I) treatment and follow-up.

**Methods:** We retrospectively reviewed 10,482 patients with DTC who underwent postoperative ^131^I treatment from 2007 to 2017 at Shanghai Sixth's People's Hospital. The relationships between progression-free survival (PFS) and several variables were assessed by univariate and multivariate analyses using the Kaplan–Meier method and a Cox proportional hazards model, respectively.

**Results:** Forty-seven patients with PTC were enrolled in this study (4.48‰ of all patients with DTC). The median age at the initial diagnosis of lung metastasis was 39.6 ± 15.4 years, and the patients comprised 14 male and 33 female patients (male: female ratio = 1.00:2.36). Twenty-five patients had ^131^I avidity and 22 had non-^131^I avidity. At the end of the 5-years follow-up, 12 patients exhibited progressive disease (PD), and 2 patients had died. At the end of the 10-years follow-up, 21 patients showed PD and five patients had died. The 5- and 10-year PFS rates were 74.47 and 53.32%, respectively; the 5- and 10-years overall survival (OS) rates were 95.74 and 89.36%, respectively. The timing of diagnosis of lung metastases, maximal size of lung metastases, and ^131^I avidity were significantly associated with the 5-years PFS rate (*P* = 0.035, *P* = 0.030, and P<0.001, respectively). Only ^131^I avidity was associated with the 10-years PFS rate (*P* < 0.001). The multivariate analyses also showed that non-^131^I avidity were the independent poor prognostic factors for 10-years PFS at the end of follow-up (*P* < 0.001).

**Conclusions:** Lung metastases from PTC in patients with persistently negative Tg and elevated TgAb levels had an excellent prognosis and survival rate during ^131^I treatment and follow-up. The loss of ^131^I avidity remained the strongest independent predictor of a poor prognosis and survival in these patients.

## Introduction

Papillary thyroid cancer (PTC) is the most common histological type of differentiated thyroid cancer (DTC), accounting for 90% of thyroid cancers (1). According to the National Cancer Center, PTC is one of the fastest growing malignant tumors in China (2), especially in women under 30 years of age, and has become the most commonly diagnosed cancer. The dramatic rise in thyroid cancer among younger women is similar to the situation in Western countries (3). Despite the increased incidence, patients with PTC usually have a good prognosis with a 5-years overall survival (OS) rate of >98.1% and 10-years OS rate of >85% to 90% from the initial diagnosis (4). However, patients with PTC have a worse prognosis in the presence of distant metastases and/or local recurrence, which seriously affect patients' quality of life and survival time (5).

Distant metastatic lesions from DTC are usually seen in the lungs, followed by the bones. During the past 70 years, radioactive iodine (^131^I) therapy has been the mainstream and routine treatment strategy for patients with DTC with lung metastasis. During the ^131^I treatment period, two-thirds of patients with DTC with lung metastases underwent therapeutic ^131^I whole-body scanning (^131^I-WBS), and half of these patients achieved remission; the remaining one-third of the patients did not have ^131^I uptake capacity and died prematurely of radioiodine-refractory DTC (RR-DTC) (6). Two recent randomized, multicenter, double-blind, placebo-controlled clinical trials showed significant improvement in progression-free survival (PFS) in patients with progressive RR-DTC treated with sorafenib and lenvatinib, which have been approved by the US Food and Drug Administration for the treatment of progressive RR-DTC (7). Several independent prognostic factors associated with a poor prognosis in patients with DTC with lung metastases have been reported, such as age at the initial diagnosis of lung metastases, ^131^I avidity, timing of diagnosis of lung metastases, maximal size of lung metastases at diagnosis, pathological type, and the presence of extrapulmonary distant metastases (8–11).

The serum thyroglobulin (Tg) level is the most sensitive and reliable marker indicating persistent or recurrent disease during follow-up after total or near-total thyroidectomy and ^131^I remnant ablation in patients with DTC. However, several factors may lead to false-negative Tg findings; e.g., a detectable amount of Tg cannot be released, the ability to secrete Tg is lost, structural changes occur in Tg, or artificially low Tg levels are induced by elevated circulating Tg antibody (TgAb) (12, 13). Previous studies have shown that TgAb interferes with serum Tg detection in 10–25% of patients with PTC with positive TgAb, especially in patients with increased TgAb levels (14). In this regard, several studies have demonstrated that a progressively increasing TgAb titer can be used as a surrogate marker for predicting persistent or recurrent disease in patients with PTC with a negative serum Tg level (15, 16). As reported in the current American Thyroid Association guideline, the criteria for negative Tg in patients with DTC are a stimulated Tg level of <10 ng/mL before ^131^I ablation, a Tg level of 0.2 ng/mL after thyroid-stimulating hormone (TSH) suppression, or a stimulated Tg level of <1 ng/mL during follow-up (3).

In clinical practice, patients with PTC with lung metastases very rarely have persistently negative Tg and elevated TgAb levels during ^131^I treatment and follow-up. To the best of our knowledge, only Viola et al. (17) has described a 56-year-old PTC patient with lung metastases who had elevated TgAb and negative serum Tg levels 10 years after initial treatment involving total thyroidectomy and ^131^I remnant ablation. Therefore, little is known about the clinical outcomes and prognostic factors of these patients with PTC. To improve our understanding of the natural history of lung metastases in these patients with PTC after total or near-total thyroidectomy with persistently negative Tg and elevated TgAb levels during ^131^I treatment and follow-up, we evaluated these patients' clinical characteristics, clinical outcomes, and independent prognostic factors in a large referral ^131^I treatment center in China.

## Materials and Methods

### Patients

**Figure 1:**
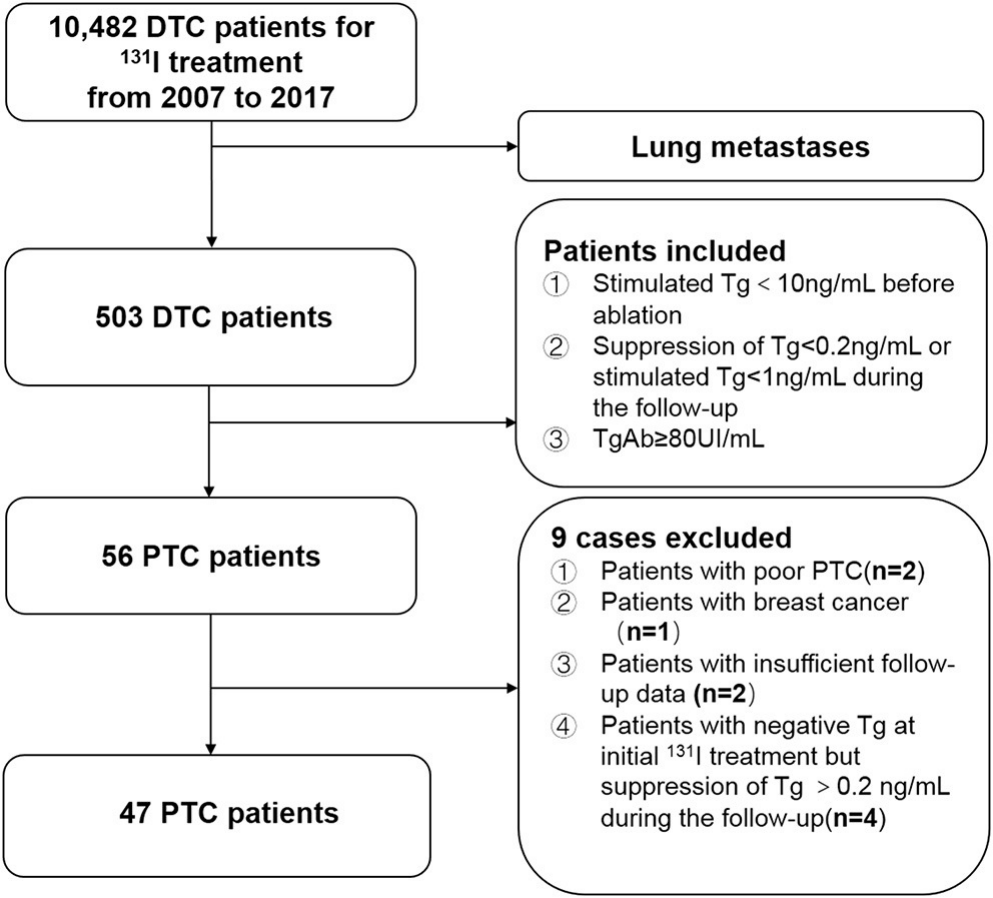
Flow chart of patients included in the current study.

We retrospectively reviewed the medical records of 10,482 consecutive patients with DTC who underwent ^131^I treatment at Shanghai Sixth's People's Hospital after total or near-total thyroidectomy from 2007 to 2017. Of these, the clinical follow-up data of 503 patients with DTC diagnosed with lung metastasis were evaluated. The inclusion criteria for patients with PTC with lung metastases who had persistently negative Tg and elevated TgAb levels during ^131^I treatment and follow-up were (i) histological confirmation of PTC, (ii) treatment with ^131^I after total or near-total thyroidectomy, (iii) a negative Tg level [defined as a preablative stimulated Tg level of <10 ng/mL at the time of initial ^131^I therapy or a Tg level of <0.2 ng/mL (TSH suppression) or <1 ng/mL (after stimulation) 6 months after the first remnant ablation according to the American Thyroid Association guideline (3)], and (iv) an elevated TgAb level (defined as a TgAb level of ≥80 UI/mL) at the time of diagnosis of lung metastasis from PTC. According to these criteria, 56 patients were identified as having lung metastases from PTC with negative Tg and elevated TgAb levels. Among them, nine patients were excluded from the current study because of poor PTC confirmed by pathological examination at the initial surgery (*n* = 2), breast cancer that may lead to lung metastases during follow-up (*n* = 1), insufficient follow-up data (*n* = 2), and a negative preablative stimulated Tg level at the time of initial ^131^I therapy but a TSH-suppression Tg level of >0.2 ng/mL during follow-up (*n* = 4) (Figure 1).

### Collection of Variables

The surgical and histopathologic data evaluated in this study were sex, maximum primary tumor diameter, pathology of primary tumor, number of primary tumors, pathological examination showing Hashimoto's thyroiditis, extrathyroidal invasion, and N stage. Among these, the maximum diameter, N stage, and extrathyroidal invasion of the primary tumor were evaluated according to the 8th edition of the TNM classification system (18). The maximum tumor diameter was divided into <2 cm, 2–4 cm, and >4 cm; the N stage was divided into N0, N1, and N2; and extrathyroidal invasion was divided into none, minimal, and gross. Poor PTC was excluded from the study, and the PTC pathology was divided into classic PTC and follicular variant of PTC.

According to the recommendation in the 7th and 8th editions of the TNM classification system (18), the cut-off age for risk stratification was set at 45 and 55 years, respectively, when lung metastases of PTC were diagnosed.

The following data regarding lung metastases from PTC were collected: timing of diagnosis of lung metastases, maximal size of lung metastases at diagnosis (mm), and ^131^I avidity. The timing of discovery of lung metastases of PTC was divided into lung metastases at presentation and delayed lung metastases. The discovery of lung metastases at presentation was defined as the detection of lung metastasis within 6 months before and after initial thyroidectomy, and the discovery of delayed lung metastases was defined as the detection of lung metastasis ≥6 months after initial thyroidectomy (19). According to the maximal diameter of the lung nodules on chest computed tomography (CT), pulmonary metastatic lesions were classified as lung nodules of >1 cm, 0.5–1 cm, or <0.5 cm. ^131^I avidity for lung metastases of PTC was defined as visible ^131^I uptake on ^131^I-WBS after ^131^I treatment, and non-^131^I avidity was defined as negative ^131^I-WBS results after ^131^I treatment or ^131^I uptake of <10% of multiple lung metastatic lesions seen on ^131^I-WBS combined with ^131^I single-photon emission CT/CT(^131^I-SPECT/CT) (20).

### Diagnostic Criteria for Lung Metastases in Patients With PTC

The diagnosis of lung metastases from PTC was established based on clinical symptoms, serum TgAb levels, chest CT findings before or after ^131^I treatment, and ^131^I avidity on therapeutic ^131^I-WBS and/or ^131^I-SPECT/CT. The diagnosis of lung metastases was confirmed by one of the following four approaches: Criterion 1, pathological puncture results confirmed lung metastasis of PTC under CT guidance before ^131^I treatment; Criterion 2, lung ^131^I avidity was seen on therapeutic ^131^I-WBS, and pulmonary nodes were found on chest CT and/or therapeutic ^131^I-SPECT/CT in patients with PTC with an elevated serum TgAb level after ^131^I treatment; Criterion 3, lung ^131^I avidity could be detected on therapeutic ^131^I-WBS, but the chest CT findings were negative and the serum TgAb level was elevated before ^131^I treatment (21); and Criterion 4, positive pulmonary nodules could be detected on chest CT in PTC patients with non-^131^I-avid lung metastases, and the TgAb level increased continuously for at least 2 years during follow-up.

### ^131^I Treatment of Lung Metastasis

At 1–6 months after total or near-total thyroidectomy combined with neck lymph node dissection, the standard ^131^I treatment at our institution was performed in these patients with PTC. Before ^131^I treatment, each patient was given a low-iodine diet and began levothyroxine withdrawal for at least 2 weeks to achieve a TSH level of ≥30 mIU/L. The patients underwent routine measurements of free triiodothyronine (FT3), free thyroxine (FT4), TSH, Tg, and TgAb; neck ultrasonography (US); and CT scans before oral administration of ^131^I treatment. We subsequently used an empirical active regimen to determine the prescribed activity of ^131^I. Specifically, 3.7 GBq (100 mCi) was taken orally for the first time to ablate thyroid remnants; oral doses of ^131^I with a standard activity of 5.55–7.40 GBq (150–200 mCi) were used for subsequent treatment of lung metastases of PTC. At 4–7 days after ^131^I oral administration, ^131^I-WBS and/or ^131^I-SPECT/CT fusion imaging was performed for these patients. The repeated ^131^I treatment was suspended in patients with non-^131^I-avid lung metastases on the ^131^I-WBS after completion of residual thyroid tissue ablation.

### Tg and TgAb Measurement

The FT3, FT4, TSH, Tg, and TgAb levels were measured before ^131^I treatment and during follow-up. A cobas® analyzer (Roche Diagnostics GmbH, Basel, Switzerland) was used to determine the Tg and TgAb levels using the same high-sensitivity electrochemiluminescence immunoassay method in the same laboratory of our hospital based on the manufacturer's instructions. Quality control was ensured by assaying the Tg and TgAb levels in control sera in each analytical series, and all sera in which the interassay coefficient of variation exceeded 10% were reassessed. The analytical limit of Tg was 0.1 μg/mL with a detection range of 0.1 to 25,000 μg/L, and the analytical limit of TgAb was 10 UI/mL with a detection range of 10 to 4,000 IU/mL. A persistently negative Tg level was defined as a preablative stimulated Tg level of <10 ng/mL at the time of initial ^131^I therapy or a Tg level of <0.2 ng/mL (TSH suppression) or <1 ng/mL (after stimulation) during follow-up (3). An elevated TgAb level was defined as a TgAb level of ≥80 IU/mL according to a previous report (22). TgAb levels were classified into three categories in this study: <1,000 IU/mL, 1,000–4,000 IU/mL, an≥4,000 IU/mL.

### Evaluation of Change in Pulmonary Nodules Based on Chest CT Imaging

Progressive disease (PD) was evaluated based on the change in metastatic lung nodules compared with baseline using chest CT according to the Response Evaluation Criteria in Solid Tumors (RECIST v1.1) during ^131^I treatment and follow-up and similar to our previous studies (19, 20). Chest CT examinations were performed with a helical CT scanner on suspended full inspiration with a slice thickness of 3 mm. The evaluation criteria were complete response (CR), defined as disappearance of all detectable target lesions; partial response (PR), defined as a 30% decrease in the sum of all target lung metastatic lesion volumes; PD, defined as a 20% increase in the sum of all lung metastatic lesion volumes, or the appearance of more than one new lesion; and stable disease (SD), defined as neither PR nor PD.

### Follow-Up

After the initial ^131^I ablation and treatment, the patients took levothyroxine orally to suppress the TSH level to <0.1 μg/mL. Regular follow-up was performed, including measurement of FT3, FT4, TSH, Tg, and TgAb; neck US; and chest CT. During the follow-up period, the FT3, FT4, TSH, Tg, and TgAb levels were measured and neck US was performed every 3–6 months, and chest CT was performed every 12 months. At the end of follow-up, OS and PFS were assessed. The OS was defined as the time from the detection of lung metastatic lesions of PTC to death of any cause, and PFS was defined as the time from the diagnosis of lung metastatic lesions of PTC to the detection of PD or death of any cause.

### Statistical Analysis

Continuous variables are presented as mean, standard deviation (SD), minimum, and maximum, and categorical variables are presented as number with percentage. The Kaplan–Meier method was applied to calculate the PFS rate of patients with PTC from the date of diagnosis of lung metastasis to the date of PD during follow-up. The 5- and 10-years PFS rates were analyzed with the Kaplan–Meier method, and differences in PFS were compared by the log-rank test with a *P* < 0.05 regarded as statistically significant. A Cox proportional hazards model was used for the multivariate analysis to investigate the associations between prognostic factors and PFS at the end of follow-up; again, a *P* < 0.05 was considered statistically significant. Statistical analyses were performed using MedCalc software version 17.0 (MedCalc Software, Mariakerke, Belgium) and GraphPad Prism 7 (GraphPad Software, San Diego, CA, USA).

## Results

### Patient Characteristics

Table 1 shows the clinicopathologic characteristics of the cohort. Among all 10,482 patients with DTC, 47 (4.48‰) patients with PTC with lung metastases who had persistently negative Tg and elevated TgAb levels were enrolled in this study. Among them, the mean age at diagnosis of lung metastases was 39.6 ± 15.4 years (median, 36 years; range, 12–70 years); 7 (14.89%) patients were ≥55 years of age, and 16 (34.04%) patients were ≥45 years of age. Of the 47 patients, 14 (29.79%) were male and 33 were female (male: female ratio = 1.00:2.36). With respect to the pathological type of primary tumor, 45 (95.74%) patients had classic PTC and 2 had the follicular variant of PTC.

**Table 1:** Clinical characteristics of the cohort.

	**Value or *N***	**%**
Age at the diagnosis of lung metastases (Year) (Mean ± SD, Median, Range)	39.6 ± 15.4, 36, 12–70	
<55	40	85.11
≥55	7	14.89
**Age at the diagnosis of lung metastases (year)**		
<45	31	65.96
≥45	16	34.04
**Sex**		
Male	14	29.79
Female	33	70.21
Maximal primary tumor size (cm) (Mean ± SD, Median, Range)	2.8 ± 0.9, 2.7, 1.5–5.2	
<2	11	23.40
2–4	30	63.83
≥4	6	12.77
**N stage**		
N0	0	0
N1a	14	29.79
N1b	33	70.21
**Pathological type**		
Classic PTC	45	95.74
FVPTC	2	4.26
**Tumor multifocality**		
No	31	65.96
Yes	16	34.04
**Extrathyroidal extension**		
No	26	55.32
Minimal	12	25.53
Gross	9	19.15
**Hashimoto's thyroiditis**		
No	34	72.34
Yes	13	27.66
**Local recurrence before the diagnosis of lung metastases**		
No	34	72.34
Yes	13	27.66
**The timing of diagnosis of lung metastases**		
At initial presentation	22	46.81
Delayed lung metastases	25	53.19
Maximal size of the lung metastases at diagnosis (mm) (Mean ± SD, Median, Range)	6.0 ± 4.4,5.0,0-22	
<5	21	44.68
5–10	19	40.43
≥1	7	14.89
^**131**^**I avidity**		
No	22	46.81
Yes	25	53.19
**TgAb level at initial** ^**131**^**I treatment (IU/mL)**		
<1,000	27	57.45
1000-4000	13	27.66
≥4,000	7	14.89
Cumulative dose of additional ^131^I activities (mCi) (Mean ± SD, Median, Range)	426.6 ± 277.2, 300, 250–1,450	
Number of courses for ^131^I therapy (Mean ± SD, Median, Range)	2.8 ± 1.5, 2.0, 2–9	
The time interval between the first and last ^131^I therapy (Year) (Mean ± SD, Median, Range)	1.1 ± 1.1, 0.5, 0.5–5.5	
Follow-up since the diagnosis of lung metastases (Year) (Mean ± SD, Median, Range)	6.9 ± 2.9, 7.4, 2–10	

### Clinical Symptoms and Diagnosis of Lung Metastases

Among all 47 patients, 4 were diagnosed based on Criterion 1, 19 were diagnosed based on Criterion 2, 3 were diagnosed based on Criterion 3, and 21 were diagnosed according to Criterion 4 One patient had synchronous bone and lung metastases, and three patients had synchronous lung and mediastinal metastases. Thirteen patients had locoregional recurrent disease without undergoing surgical resection; of these 13 patients, 10 had a solitary lymph node metastasis, the size of which stabilized during follow-up, and the patients were unwilling to undergo surgery. The remaining three patients had locally invasive lesions that could not be surgically resected. Forty-three patients with lung metastases were asymptomatic, while four had clinical symptoms related to the lung metastatic lesions: breathing difficulty in one, hemoptysis in one, and dry cough in two.

### Changes in Serum TgAb Levels

**Figure 2:**
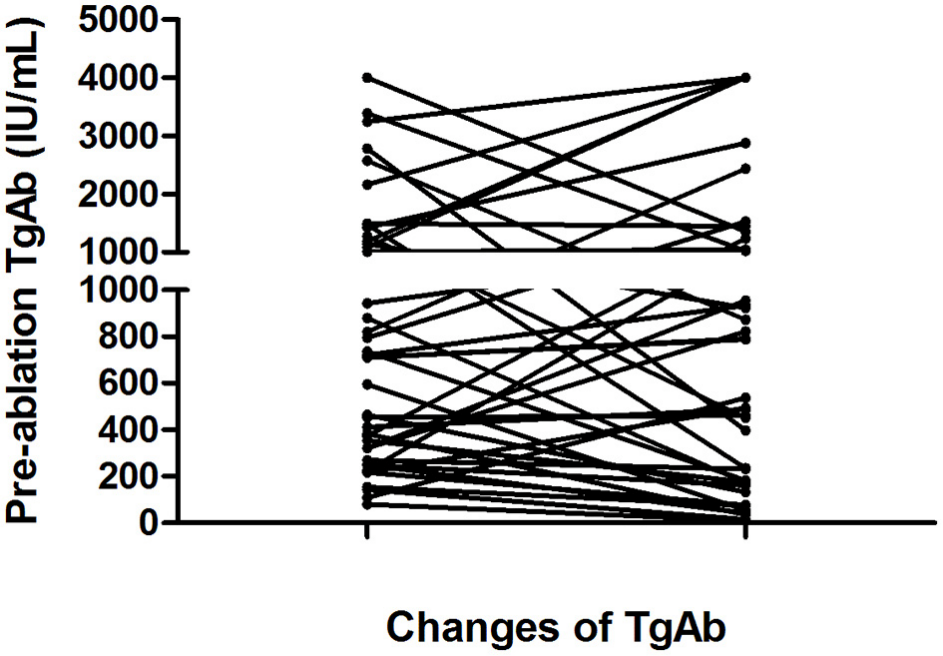
Changes in serum TgAb levels from the initial ^131^I treatment to the end of follow-up in 41 of the 47 patients with persistently negative Tg and elevated TgAb levels. Six patients were excluded from this figure because their TgAb levels were consistently higher than 4,000 IU/mL during ^131^I treatment and follow-up.

According to the serum TgAb grouping method used in the present study, the TgAb level was <1,000 IU/mL in 27 (57.45%) patients, 1,000–4,000 IU/mL in 13 patients, and >4,000 IU/mL in 7 patients. The changes in the serum TgAb levels are shown in Figure 2.

In the <1,000 IU/mL group, the serum TgAb level decreased in 25.53% (12/47) of patients with an average change of 373 (range, 81–880) to 94.5 (range, 10–182) IU/mL from the diagnosis of pulmonary metastases to the end of follow-up. The Tg level increased in the remaining 31.91% (15/47) of patients with an average change of 493.6 (range, 109–944) to 917.7 (range, 232–2,435) IU/mL.

In the 1,000–4,000 IU/mL group, a decrease in the serum TgAb level was seen in seven (14.9%) patients and an increase was seen in six (12.8%). In the seven patients with a decreased TgAb level, the average TgAb level decreased from 2,021 (range, 1,496–3,391) to 766 (range, 236–1,452) IU/mL. In the six patients with an increased TgAb level, the mean TgAb level was 1687.5 IU/mL (range, 1,008–3,245 IU/mL) before ^131^I treatment; at the end of follow-up, five (10.6%) patients' TgAb level had increased to 4,000 IU/mL, and one patient's TgAb level had increased from 1,008 to 1,048 IU/mL.

In the >4,000 IU/mL group, only one patient's serum TgAb gradually decreased from >4,000 to 1,356 U/mL, and in the remaining six patients, the serum TgAb levels were still >4,000 IU/mL after ^131^I treatment and follow-up.

### Treatment Outcome of Lung Metastases

All 47 patients with PTC patients with lung metastases who had persistently negative Tg and elevated TgAb levels during follow-up underwent ^131^I treatment. The ^131^I treatment outcome were showed in Table 1. Twenty-five (53.19%) patients had ^131^I avidity and 22 (46.81%) had non-^131^I avidity on ^131^I-WBS after an oral therapeutic dose of ^131^I. Among the 25 patients with ^131^I uptake of lung metastases, diffuse absorption was seen on ^131^I-WBS in 3 patients with elevated TgAb levels after ^131^I treatment, but lung nodules could not be detected by chest CT. Of the 22 patients with non-^131^I avidity, only 1 patient who developed progressive thyroid cancer was treated with sorafenib. Sorafenib was administered at a dose of 200 mg orally twice a day. A cycle was defined as 4 weeks. This patient developed a severe hand–foot reaction and stopped taking the sorafenib after three cycles. Neither chemotherapy nor external beam irradiation was used to treat the lung metastases in this study.

### Results of 5- and 10-Years OS and PFS

**Figure 3:**
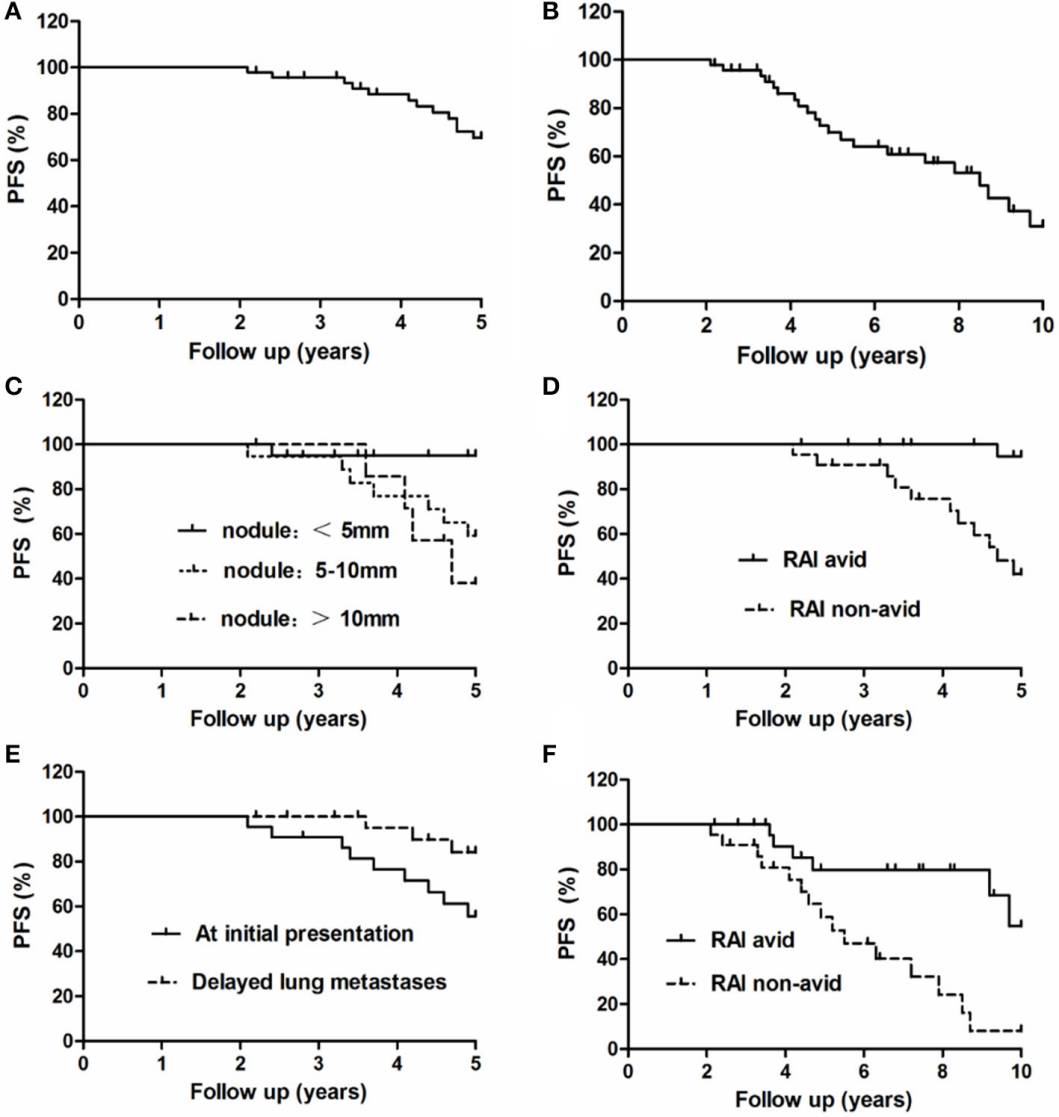
Progression-free survival of all 47 patients with lung metastases at the end of the **(A)** 5-years and **(B)** 10-years follow-up. Comparison of PFS curves for these 47 patients with lung metastases at the end of the 5-years follow-up according to the **(C)** maximal size of lung nodules, **(D)** ^131^I avidity, and **(E)** timing of metastasis. Comparison of PFS curves for these 47 patients with lung metastases at the end of the 10-years follow-up according to the **(F)** ^131^I avidity.

The follow-up time ranged from 2 to 10 years (mean, 6.9 ± 2.9 years). At the end of follow-up, 42 patients were alive and 5 had died. Of the five patients who died, one died of locally recurrent disease 2.4 years after the diagnosis of lung metastasis; two died of lung metastasis at 5.5 and 9.1 years after the diagnosis of lung metastasis, respectively; and two died of cardiac disease and myeloma at 4.1 and 9.4 years from the initial follow-up, respectively. Based on the RECIST v1.1 criterion using chest CT imaging, at the end of the 5-years follow-up, seven patients had obtained CR, including three with undetectable pulmonary nodules on chest CT; eight patients had PR; 20 had SD; and 12 had PD. At the end of the 10-year follow-up, seven patients had achieved CR, seven had PR, 12 had SD, and 21 had PD. The 5- and 10-years OS rates were 95.74 and 89.36%, respectively, and the 5- and 10-years PFS rates were 74.47 and 53.32%, respectively (Figures 3A,B).

### Factors Predicting 5- and 10-Year PFS Rate

The 5-years PFS rate and its prognostic factors in the 47 patients with lung metastases as evaluated by the Kaplan–Meier method and log-rank test are listed in Table 2. At the end of the 5-years follow-up period, 12 patients exhibited PD and 35 did not. In the univariate analysis, the timing of diagnosis of lung metastases, maximal size of lung metastases, and presence of ^131^I avidity were significantly associated with the 5-years PFS rate. Patients with lung metastatic lesions of <5 mm, delayed lung metastases, and ^131^I-avid disease had higher 5-years PFS rates than patients with lung metastatic lesions of 5–10 mm/≥10 mm, lung metastases at initial presentation of PTC, and non-^131^I-avid disease, respectively. Nevertheless, other several factors were not significantly associated with the 5-years PFS rate (Figures 3C–E).

**Table 2:** Univariate analysis of significant prognostic factors influencing 5-years PFS rate in all 47 patients.

**Variable**	**No. of patient**	**5-years**		**Log-rank value**	***P***
		**No. of PD**	**PFS rate (%)**		
Age at the diagnosis of lung metastases (Year)				2.378	0.123
<55	40	9	77.50		
≥55	7	3	57.14		
**Age at the diagnosis of lung metastases (Year)**					
<45	31	7	77.42	0.773	0.379
≥45	16	5	68.75		
Sex				0.295	0.587
Male	14	3	78.57		
Female	33	9	72.73		
Maximal primary tumor size (cm)				2.749	0.253
<2	11	1	90.91		
2–4	30	10	66.67		
≥4	6	1	83.33		
**N stage**					
N1a	14	3	78.57	0.127	0.721
N1b	33	9	72.73		
Tumor multifocality				0.134	0.715
No	31	7	77.42		
Yes	16	5	68.75		
Extrathyroidal extension				5.264	0.072
No	26	4	84.61		
Minimal	12	3	75.00		
Gross	9	5	44.44		
Hashimoto's thyroiditis				0.819	0.366
No	34	10	70.59		
Yes	13	2	84.62		
Local recurrence before the of lung diagnosis				3.400	0.065
No	34	6	82.35		
Yes	13	6	53.85		
The timing of diagnosis of lung metastases				4.468	0.035
At initial presentation	22	9	59.09		
Delayed lung metastases	25	3	88.00		
Maximal size of lung metastases at diagnosis (mm)				7.156	0.030
<5	21	1	95.24		
5–10	19	7	63.16		
≥10	7	4	45.86		
^131^I avidity				13.86	<0.001
Yes	25	1	96.00		
No	22	11	50.00		
TgAb level at initial ^131^I treatment (IU/mL)				0.641	0.725
<1,000	27	7	74.07		
1,000–4,000	13	3	76.92		
≥4,000	7	2	71.43		

The 10-years PFS rate and its prognostic factors in the 47 patients with lung metastases as evaluated by the Kaplan–Meier method and log-rank test are listed in Table 3. At the end of the 10-years follow-up period, 21 patients showed PD; the univariate analysis demonstrated that only ^131^I avidity was associated with 10-years PFS rate in these patients. Patients without ^131^I avidity had more significantly shorter than patients with ^131^I avidity. However, there was no significant association between the 10-years survival rate and the remaining several factors (Figure 3F).

**Table 3:** Univariate analysis of significant prognostic factors influencing 10-years PFS rate in all 47 patients.

**Variable**	**No. of patient**	**10-years**		**Log-rank value**	***P***
		**No. of PD**	**PFS rate (%)**		
Age at the diagnosis of lung metastases				0.007	0.933
<55	40	18	55.00		
≥55	7	3	57.14		
Age at the diagnosis of lung metastases				0.838	0.360
<45	31	13	58.06		
≥45	16	8	50.00		
Sex				0.135	0.713
Male	14	7	50.00		
Female	33	14	54.84		
Maximal primary tumor size (cm)				0.611	0.737
<2	11	5	54.55		
2–4	30	13	56.67		
≥4	6	3	50.00		
N stage				0.003	0.864
N1a	14	7	50.00		
N1b	33	14	57.58		
Tumor multifocality				1.301	0.254
No	31	11	64.52		
Yes	16	10	37.50		
Extrathyroidal extension				1.016	0.602
No	26	11	57.69		
Minimal	12	5	58.33		
Gross	9	5	44.44		
Hashimoto's thyroiditis				0.902	0.342
No	34	15	55.88		
Yes	13	6	53.85		
Local recurrence before PM diagnosis				0.278	0.598
No	34	14	58.82		
Yes	13	7	46.15		
The timing of diagnosis of lung metastases				0.370	0.543
At initial presentation	22	10	54.55		
Delayed lung metastases	25	11	56.00		
Maximal size of PM at diagnosis (mm)				3.791	0.150
<5	21	6	71.43		
5–10	19	10	47.37		
≥10	7	5	28.57		
^131^I avidity				10.81	<0.001
Yes	25	6	76.00		
No	22	15	31.82		
TgAb level at initial ^131^I treatment (IU/mL)				0.606	0.739
<1,000	27	9	66.67		
1,000–4,000	13	8	38.46		
≥4,000	7	4	43.86		

A Cox proportional hazards model was used to assess the independent predictors of the 10-years PFS rate (Table 4). The absence of ^131^I avidity (relative risk, 1048.45; 95% confidence interval, 26.600–41324.708; *P* < 0.001) were independent poor prognostic factors for the 10-years PFS rate in the multivariate analysis. Other several factors were not predictive of the 10-years PFS rate.

**Table 4:** Multivariate analysis of prognostic factors of 10-years PFS rate in all 47 patients using a Cox proportional hazards model at end of follow-up.

**Variable**	**Risk ratio**	**95% CI**	**P**
Age at the diagnosis of lung metastases			0.306
<55	1		
≥55	0.15	0.004–5.600	
Age at the diagnosis of lung metastases			0.474
<45	1		
≥55	2.46	0.213–28.299	
Sex			0.491
Male	1		
Female	1.63	0.411–6.453	
**Maximal primary tumor size**			
<2	1		
2–4	1.27	0.159–10.155	0.823
≥4	2.34	0.497–8.185	0.273
*N* stage			0.880
N1a	1		
N1b	1.18	0.137–10.179	
Tumor multifocality			0.142
No	1		
Yes	2.367	0.344–5.895	
**Extrathyroidal extension**			
No	1		
Yes	0.21	0.031–1.471	0.145
Hashimoto's thyroiditis			0.057
No	1		
Yes	3.189	0.339–4.99	
Local recurrence before the diagnosis of lung metastases			0.477
No	1		
Yes	0.38	0.026–5.482	
The timing of diagnosis of lung metastases			0.093
At initial presentation	1		
Delayed lung metastases	2.329	1.262–4.986	
**Maximal size of lung metastases at diagnosis**			
<5	1		
5–10	1.01	0.157–6.475	0.994
≥10	5.82	0.402–84.188	0.198
^131^I avidity			<0.001
Yes	1		
No	1048.45	26.600–41324.708	
**TgAb level at initial** ^**131**^**I treatment (IU/mL)**			
<1,000	1		
1,000–4,000	0.023	0.002–0.283	0.053
≥4,000	0.906	0.074–11.127	0.939

## Discussion

In the current study, we retrospectively analyzed 47 patients with PTC with lung metastases who had persistently negative serum Tg and elevated serum TgAb levels during ^131^I treatment and follow-up. The study showed that the 5- and 10-years OS rates were 95.74 and 89.36% and that the 5- and 10-years PFS rates were 74.47 and 53.32%, respectively. Lung metastatic lesions of 5–10 mm/≥10 mm, lung metastases at initial presentation of PTC, and non-^131^I-avid lung metastases predicted a poor 5-years PFS rate, and only patients without ^131^I avidity showed a poor 10-years PFS rate. The absence of ^131^I avidity were the independent poor prognostic factors for the 10-years PFS rate at the end of follow-up.

The median age of the 47 patients with PTC with lung metastases at diagnosis was 39.6 years (range, 12–70 years), which is significantly lower than that reported in patients with DTC with lung metastases. Previous studies have shown that the median age of patients with DTC with lung metastases varies from 45 to 52 years (8–11, 21, 23, 24). Age has been recognized as a predictive factor that is closely associated with survival and recurrence in patients with DTC with lung metastases, but this remains controversial (25, 26); however, most studies have suggested that age can be used as an independent prognostic factor for DTC (8–11, 21, 23, 24, 26–28). The cut-off age for risk stratification was set at 45 and 55 years according to the recommendations of the 7th and 8th editions of the TNM classification system, respectively (18). In the present study, 7 (14.89%) patients were ≥55 years of age and 40 patients were <55 years of age, while 16 (34.04%) patients were ≥45 years of age, and 31 patients were <45 years of age. The log-rank test showed that a cut-off age of 55 or 45 years was not significantly associated with the 5- and 10-years PFS rates in these patients in the univariate analysis. Study have showed that a cut-off age of 55 years resulted in higher accuracy for prognosis and recurrence in patients with DTC than a cut-off age of 45 years (18, 29); however, the cut-off of 55 years did not provide advantages over the cut-off of 45 years in the present study. A possible explanation is that the sample size of patients aged >55 years was too small for a statistical analysis, producing a deviation (patients ≥55 vs. <55 years: ratio of 1.0:5.7). Our multivariate analysis also showed that age was not an independent prognostic factor for these patients.

In previous reports, the incidence of non-^131^I-avid lung metastases from DTC ranged from 5 to 45%, with an average value of about 30% (8–11, 21, 23, 24). In the present study, ^131^I was not absorbed by lung metastases in 46.81% (22/47) of patients with PTC with persistently negative Tg and elevated TgAb levels; this incidence is clearly higher than that reported above. This might also suggest differences in sample sizes, selection bias, and/or screening methods used in the referral population in our center and/or in clinical management protocols after DTC diagnosis; all of these factors affect the incidence rate of non-^131^I avidity in patients with DTC. ^131^I avidity is well-recognized as a prognostic factor for lung metastases from DTC. Several studies have also indicated that DTC patients with ^131^I avidity showed longer OS, disease-free survival, or PFS than those with non-^131^I-avid lung metastases (8–11, 21, 23, 24). Consistent with previous studies, our study showed that the 5- and 10-years PFS rates were significantly higher at the end of follow-up in patients with than without ^131^I avidity (96.00 vs. 50.00% and 76.00 vs. 31.82%, respectively). The multivariate analysis also showed that ^131^I avidity is the strongest predictive factor for a better prognosis in these patients.

Normally, when the largest lung metastatic lesion of DTC measures <1 cm, it is defined as a micronodule, and a >1-cm lesion is defined as a macronodule. Most studies have confirmed that lung metastases composed of micronodules are an important predictor of a better prognosis and longer survival in patients with DTC; studies have shown that compared with macronodules, micronodules are associated with significantly improved OS or PFS in patients with DTC (10, 11, 30). However, the ratio of micronodules to micronodules and its association with prognosis and survival are not inconsistent among previous studies For example, Sohn et al. (11) showed that among 89 patients with only lung metastases who were treated from 1996 to 2012, the ratio of micronodules to macronodules was 1.00:2.33 (24/56) with the 10-years PFS rate for about 60% and 28%, respectively. Mona et al. (23) reported that among 199 consecutive patients with follicular cell-derived thyroid cancer with lung metastases who underwent ^131^I treatment, 75% had a <1-cm nodule as the largest lesion and 25% of patients had a ≥1-cm nodule as the largest lesion, producing a ratio of 3:1 and the 10-years PFS rate was about 37 and 5%, respectively. These differences may be due to the different criteria used to select patients and the different modes of diagnosis and treatment. In the present study, the size of the lung metastases in 40 patients was <1 cm and that in seven patients (14.89%) was >1 cm, for a ratio of 5.71:1.00. Because the number of patients with micronodules and macronodules varies considerably, statistical deviations may occur. To avoid this, we classified the lung metastases as lung nodules of >1 cm, 0.5–1 cm, and <0.5 cm. Our study showed that the size of the lung nodule could affect the 5-years PFS rate but could not affect 10-years PFS rate. The multivariate analysis also showed that the size of the largest lung metastatic lesion was not an independent prognostic factor at the end of the 10-years follow-up.

The presence of distant metastatic disease at presentation is relatively rare, with a rate of 3% to 15%. In our study, 22 (46.81%) patients had lung metastases at the initial presentation, and 25 (53.19%) had delayed lung metastases during follow-up. The presence of lung metastatic disease at presentation was higher in our series than in past reports. Some investigators have reported that delayed metastasis has a worse prognosis than early metastasis (10, 11, 31). In contrast, our study showed that the 5-years PFS rate in patients with delayed metastasis was significantly higher than that in patients with lung metastasis at presentation; however, there was no significant difference in 10-years PFS rate between the two groups. This discrepancy might be explained by the difference in the number of patients with ^131^I avidity between these two groups.

Stimulated Tg assessment was performed after withdrawal TSH from thyroid replacement therapy or after stimulation with recombinant human TSH. Not only is a high stimulated Tg level of >10 μg/mL considered an indicator of recurrent/metastatic disease in patients with DTC, but a high stimulated Tg level of ≥50 μg/mL is associated with shorter PFS and OS in patients with DTC (32). Because a gradual increase in the TgAb titer after thyroidectomy can be used as a predictor of persistent or recurrent disease in patients with DTC with a negative serum Tg level (15, 16), we investigated whether high levels of TgAb are also associated with shorter PFS and OS of patients with PTC with lung metastases. In this study, the TgAb levels were classified into three categories: <1,000 IU/mL, 1,000–4,000 IU/mL, and >4,000 IU/mL, and the percentages of patients in these three groups were 57.45% (27/47), 27.70% (13/47), and 14.89% (7/47), respectively. Our study showed no significant difference in the 5- or 10-years PFS rate among the three groups. The multivariate analysis also showed that the TgAb level was not an important prognostic factor for the 10-years PFS rate among the three groups at the end of follow-up. The use of the TgAb level as a prognostic parameter in patients with DTC remains controversial. Rubello et al. (33) and Adel et al. (34) reported a higher post-ablation local recurrence rate that was associated with a persistently increased TgAb level. However, most authors found no correlation between these parameters (35, 36). To the best of our knowledge, the present study is the first to evaluate the relationship between the TgAb level and lung metastasis in these patients with PTC. Larger sample sizes and longer-term follow-up of these patients are needed for further validation.

It is well-known that about 20–30% of PTC patients have Hashimoto's thyroiditis (37–39); our study showed a 27.66% (13/47) rate PTC patients with Hashimoto's thyroiditis, which was basically accordant with the above study. Whether Hashimoto's thyroiditis is related to the prognosis in PTC patients remains controversial; some anthers have reported that PTC patients with Hashimoto's thyroiditis are associated with better TNM staging and prognosis, with a lower rate of lymph node metastases, distant metastasis and recurrence rate because of its protection (39–42). Other studies have demonstrated that PTC patients with preexisting Hashimoto's thyroiditis are closely associated with the increased risk of recurrence (37, 38, 43–45). In the present study, we found that Hashimoto's thyroiditis wasn't associated with PFS and this may be related to the relatively limited number of patients, selected deviation and different diagnostic diagnosis and treatment methods and so on.

In addition to age, ^131^I avidity and the size of lung metastases may also be associated with a better prognosis and longer survival of patients with lung metastases from DTC, including patients with DTC with only lung metastases, non-extrathyroidal extension, and so on. In the present study, the pathological type was PTC in all 47 patients because follicular thyroid carcinoma with lung metastases rarely causes the TgAb levels to rise. Studies have demonstrated that coexisting bone metastases are more closely associated with the prognosis than are solitary lung metastases of DTC (8–11). Of our 47 patients with lung metastases, bone metastases were found in only 1 patient. In our series of 47 patients with PTC, the 5- and 10-years OS rates were 95.74 and 89.36%, respectively. These survival rates are obviously higher than those of patients with DTC with lung metastases reported in previous studies (50–93% and 48–85%, respectively). In our study, only five patients had died by the end of the follow-up; therefore, the prognostic factors associated with OS in these patients were not assessed by univariate and multivariate analyses. We speculate that the better prognosis of these patients was mainly related to the above-mentioned factors (age at the diagnosis of lung metastases, ^131^I avidity, size of the lung metastases, the presence of only lung metastases, and so on).

The present study has several limitations. First, it has certain inherent limitations associated with its retrospective design. Second, we only registered patients from a single tertiary referral center, and we included a relatively limited number of patients. Nonetheless, this is the first assessment of the clinical outcome in patients with lung metastases from PTC with persistently negative Tg and elevated TgAb levels during ^131^I treatment and follow-up. Third, in all eligible patients, the lung nodules were confirmed by a CT scan, significantly increased serum TgAb levels, and ^131^I-WBS because it is unrealistic and unethical to obtain histological evidence of diffuse lung lesions in these patients. Fourth, our study lacked a control group in DTC patients with elevated Tg. In our past study, however, we reported the survival and prognosis of 372 DTC patients with lung metastases and elevated Tg, whose 10-years OS rate was significantly lower than that of PTC patients with elevated TgAb and persistently negative Tg level (10). Moreover, only a few other diseases can cause an increase in the TgAb level, such as type 1 diabetes, rheumatoid arthritis, and malignant anemia and so on (46). When PTC and multiple inflammatory lung nodules are present simultaneously, it is difficult to confirm them and they can only be excluded by follow-up, especially for non-^131^I-avid lung metastases of PTC.

## Conclusion

This study is the first to evaluate the clinical characteristics, outcomes, and prognostic factors of lung metastases from PTC in patients with persistently negative Tg and elevated TgAb levels, which represents an extremely rare pattern of invasion of PTC with an incidence of only 4.48‰. In these patients, the 5- and 10-years OS rates were 95.74 and 89.36%, respectively, and the 5- and 10-years PFS rates were 74.47 and 53.32%, respectively. These rates suggest that lung metastasis from DTC has an excellent prognosis and good survival in patients with PTC with persistently negative Tg and elevated TgAb levels during ^131^I treatment and follow-up. The timing of diagnosis of lung metastases, maximal size of lung metastases, and ^131^I avidity may be associated with the 5-years PFS rate, and only ^131^I avidity was associated with the 10-years PFS rate in these patients with lung metastases. The loss of ^131^I avidity was the strongest poor independent prognostic factor for prognosis and survival in these patients.

## Data Availability Statement

All datasets generated for this study are included in the article/supplementary material.

## Ethics Statement

The studies involving human participants were reviewed and approved by the Ethics Committee of the Sixth People's Hospital Affiliated to Shanghai Jiaotong University. Written informed consent was obtained from the individual(s), and minor(s)' legal guardian/next of kin, for the publication of any potentially identifiable images, or data included in this article.

## Author Contributions

Z-LQ and Q-YL designed the study. Z-KS and C-TS conducted the statistical analysis. G-QZ and H-JS collected the clinical data. Z-LQ wrote the whole paper. Z-KS and Q-YL supervised and edited the paper. All authors read and approved the final paper.

### Conflict of Interest

The authors declare that the research was conducted in the absence of any commercial or financial relationships that could be construed as a potential conflict of interest.
